# Pervasive liquid metal printed electronics: From concept incubation to industry

**DOI:** 10.1016/j.isci.2020.102026

**Published:** 2021-01-05

**Authors:** Sen Chen, Jing Liu

**Affiliations:** 1 Beijing Key Lab of Cryo-Biomedical Engineering, Technical Institute of Physics and Chemistry, Chinese Academy of Sciences, Beijing 100190, China; 2 School of Future Technology, University of Chinese Academy of Sciences, Beijing 100039, China; 3 Key Lab of Cryogenics, Technical Institute of Physics and Chemistry, Chinese Academy of Sciences, Beijing 100190, China; 4 Department of Biomedical Engineering, School of Medicine, Tsinghua University, Beijing 100084, China

**Keywords:** Electronic Design Automation, Metals, Electronic Materials, Devices

## Abstract

Electronic devices play vital role in modern civilization. Compared to conventional electronic manufacturing, the recently emerging liquid metal printed electronics (LMPE) is opening many extraordinary opportunities, such as large-area printing, pervasive adaptability, flexibility for personal use, low cost, high performance, and environmental friendliness. More uniquely, liquid metal printing allows customize electronic products on demand to fabricate electronics spanning from 2D plane surface to 3D structure and on any desired substrates. This deems it to reshape modern electronics and integrated circuits field. So far, a variety of technological breakthroughs in this new generation electronic engineering area have been made in the process of developing various liquid metal functional inks, printing machines and applications, which significantly stimulate the quick incubation and formation of a new electronic industry. Clearly, sorting out the major R&D directions and clarifying future challenges is crucial for the large scale industrialization of LMPE. This perspective article is dedicated to briefly outline the representative principles and key technologies lying behind, and illustrate the milestone products and equipment thus invented for the coming LMPE industry. In addition, we evaluate the corresponding industrialization trends and promising roadmap and interpret future prospects for the new era of pervasive electronics when anyone can freely use such a tool to print out himself functional electronic device to fulfill various purposes at anywhere and anytime.

## Graphical Abstract

**Figure:**
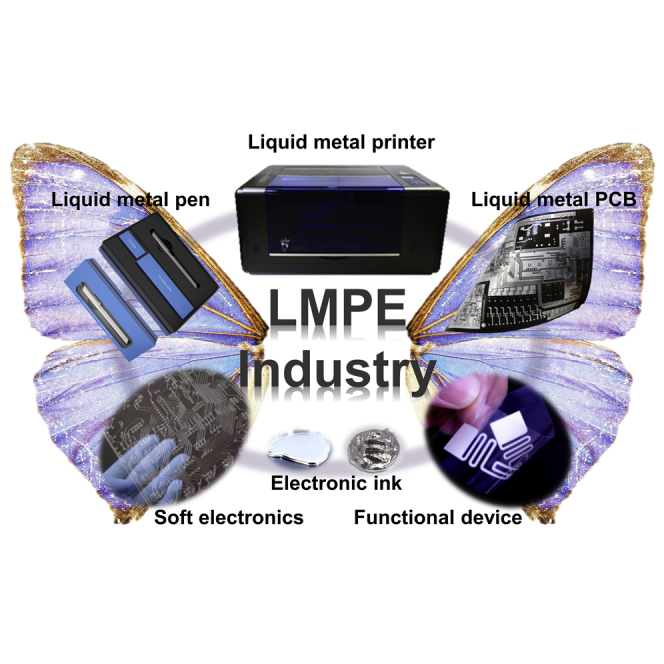

## Introduction

With the advent of the information age, electronic products are playing an ever important role. Over the past few decades, various electronic manufacturing methods have been intensively tried (Comiskey et al., 1998; Minemawari et al., 2011; Zhou et al., 2012). Among them, silicon-based semiconductor microelectronics almost occupied the absolute dominant position in the whole electronic industry. However, due to the increasing complexity of such integrated circuit (IC) fabrication and the huge investment required, the realization of silicon-based ICs is often monopolized in those hands of a few large companies throughout the world. This is definitely not beneficial for the rapid progress of pervasive electronic engineering which can be accessed by an ordinary user. Revolutionizing the traditional preparation process has then become a tough target facing current society (Sirringhaus et al., 2000; Yan et al., 2009). Meanwhile, the research and development of electronic inks consisting of classical organic and inorganic semiconductor materials have spawned the exploration of manufacturing various electronic devices through only traditional printing technology. A biggest feature of liquefied organic and inorganic semiconductor materials lies in that they do not depend on the conductor or semiconductor properties of the base material. Besides, such electronic inks can be directly deposited on desired substrate via additive manufacturing, resulting in options unlike silicon-based microelectronics. As a new alternative, printed electronics therefore achieved rather rapid progress in recent years.

**Figure 1:**
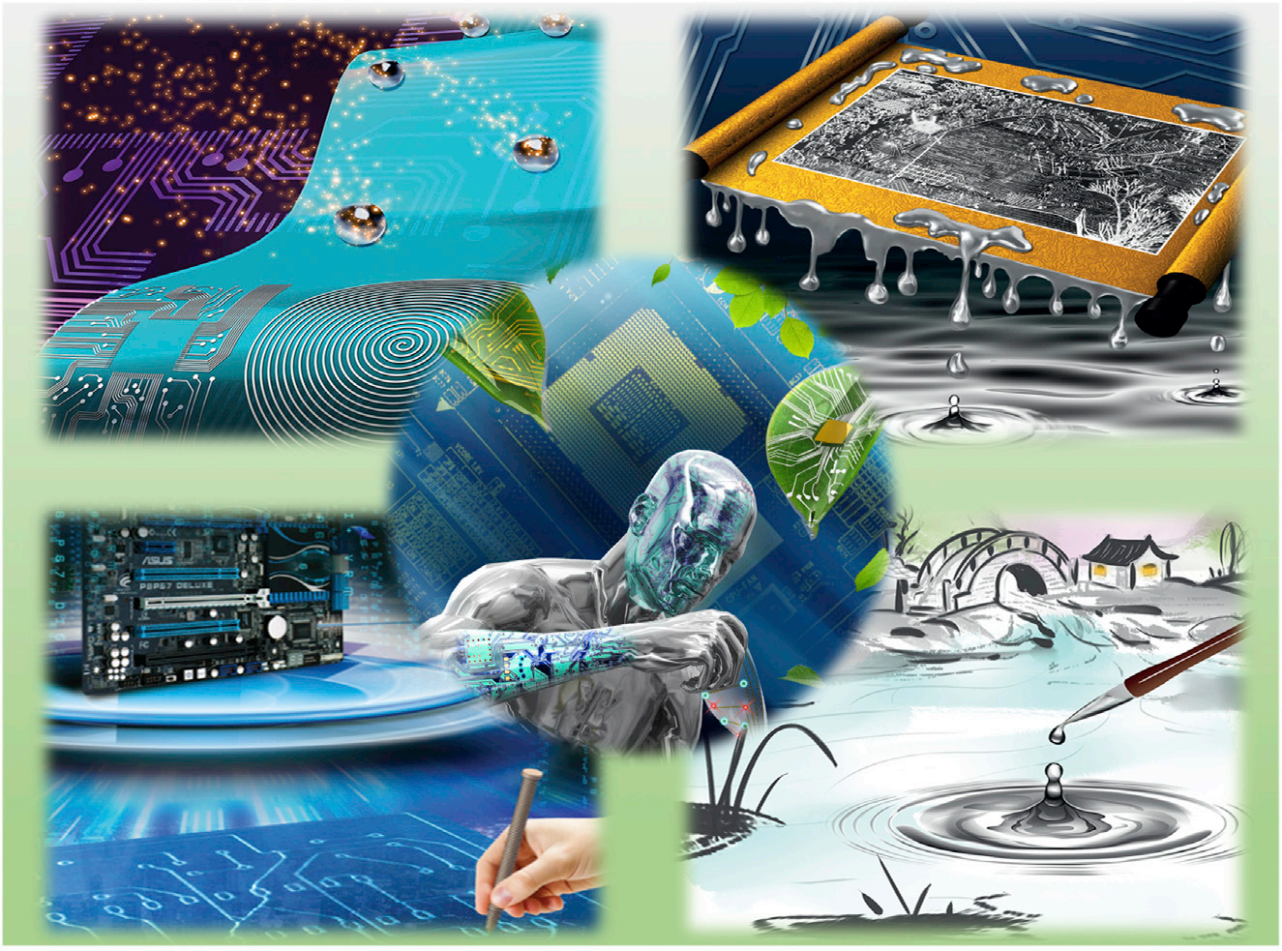
A glimpse of the rapidly developing liquid metal printed electronics

In principle, printed electronics display many outstanding advantages, such as large-area preparation, low cost and environmental friendliness, which is mainly owing to its simplified processing and material saving (Parashkov et al., 2005). Distinguishing from traditional photolithography molding process which generally requires multiple steps of gluing, exposure, development, and etching, involving a large number of vacuum processes and taking pretty long time, printed electronics basically belongs to a one-step manufacture with extremely high production efficiency (Carlson et al., 2012; Cui 2016). Up to now, the main limitation of printed electronics lies in its availability of high performance inks and the printing ways thus involved. Usually, such electronic inks include many inorganic nanomaterials (Choi et al., 2015; Jason et al., 2015; Kamyshny and Magdassi, 2019; Liu et al., 2018b; Pidcock and Panhuis, 2012) (e.g. carbon nanotubes, graphene, nanocopper, nanosilver) with excellent conductivity. Intrinsically, the electrical properties of the classical inorganic nanomaterials are only embodied in the monomer material. That is, a single carbon nanotube or a single graphene may display high electrical conductivity. However, once it was prepared into a printable ink, the electrical properties of the printed nanomaterial will be significantly reduced and many subsequent complicated processing will have to be used to realize final electrical conduction (Table 1). Further, internal particle agglomeration, chemical property denaturation throughout process and deposition often affect the ultimate performances of conductive ink, and post-processing is usually required to make the ink conductive, which are yet to be sufficiently resolved. Facing such tough situation, the recent emergence of liquid metal printed electronics (LMPE) (Zhang et al., 2012) is offering a superb opportunity to attack this bottleneck, which also becomes one major theme of the current printed electronics over the world (Liu and Wang, 2019). From both theoretical and technical aspects, such unconventional electronic manufacture strategy may finally make pervasive electronics a reality. Overall, the LMPE, as shown in Figure 1, is opening a great many opportunities from science, technology to industrialization and a wonderful future is expected to come.

**Table 1:** A comparison of several typical conductive inks

Ink type	Ink compositionρ	Conductivity	Post-processingμ
Polymer conductive ink	PEDOT:PSS	8.25 × 10^3^ S/m	150°C/20 min
Carbon conductive ink	Carbon CNT	1.8 × 10^3^ S/m 5.0 × 10^3^ S/m	Need
Nanosilver ink	Ag-DDA Ag-PVP	3.45 × 10^7^ S/m 6.25 × 10^6^ S/m	140°C/60 min 260°C/3 min
Liquid metal ink	EGaIn Ga, In, Sn, In, etc	3.4 × 10^6^ S/m	Not needed

(Pidcock and Panhuis, 2012; Xiong and Liu, 2012; Mo et al., 2011; Lee et al., 2005; Yan et al., 2018).

As is now gradually understood, liquid metal is in a large extent a most desirable choice for preparing electronic inks because of its high charge mobility (up to 10^6^ S/m), adjustable physical and chemical properties (Daeneke et al., 2018; Elton et al., 2020; Ren et al., 2020), such as low melting point, high thermal conductivity, and electrical conductivity, etc., which are illustrated in Table 2. The liquid metal mentioned here generally refers to a large class of emerging functional materials with many unique natures, such as including Ga-based liquid metal and Bibased liquid metal. Owing to its low melting point (Gong et al., 1993; Ma and Liu, 2007b; Zhang et al., 2019c), manufacturing process of LMPE does not require high-temperature condition. In addition, liquid metal is environmentally friendly, almost non-toxic (Kim et al., 2018; Shaini et al., 2000; Yan et al., 2018; Yi and Liu, 2017), and contains many novel features that have never been recognized before. Currently, it is providing significant enlightenment and extremely rich space for a huge number of emerging scientific and technological frontiers (Kalantar-Zadeh et al., 2019; Liu et al., 2019; Syed et al., 2019; Wang et al., 2018a; Wu et al., 2018a; Yan et al., 2018; Zavabeti et al., 2017; Zeng and Fu, 2018; Zhu et al., 2019; Yun et al., 2020; Ford et al., 2020). Over the long years' research experiences in liquid metal and bearing in mind current challenges facing existing printed electronics, we gradually conceived and systematically proposed a new area of LMPE that is completely different in principles from the conventional ones (Zhang et al., 2012) through introducing highly conductive inks made of low-melting metals and their alloys, signifying a big paradigm shift in technology. Along this direction, a series of fundamental principles and practical technologies in molding new generation liquid metal electronic inks and printers have been established. Among them, a group of LMPE equipment, inks and application products have been even translated into industry and a rapid industrialization is being witnessed in the area.

**Table 2:** Physical and chemical properties of liquid metals

Materials	Density (kg/m3)	Electrical conductivity (10^6^ S/m)	Viscosity (10^−3^ Kg/m·s)μ	Thermal conductivity (W/m·K)	Melting point (°C)
Hg	13,564	1.04	1.56	8.7 (at 25°C)	−38.8
Cs	1879	4.89	0.63	17.4 (at 100°C)	28.5
Ga_61_In_25_Sn_13_Zn_1_	6500	–	–	15	7.6
Ga_68.5_In_21.5_Sn_10_ (Galinstan)	6440	3.46	2.22	39	10.7
GaIn_21.4_ (EGaIn)	6280	3.4	1.99	26.81	15.4
Ga	6080	6.73	1.37	33.68 (liquid)	29.8

(Yan et al., 2018; Chen and Liu, 2019; Yi and Liu, 2017; Daeneke et al., 2018).

So far, LMPE has attracted rather widespread attention spanning from academia to industry, demonstrating an ever increasingly bright future. This perspective article is dedicated to present a brief introduction about the developmental roadmap of LMPE and interpret the core issues toward further large scale industrialization. Challenges and future directions worth of pursing will be discussed, hoping to provide a general view for the sustainable development of the coming LMPE industry.

## Basic principles and key technologies

With completely new generation electronic inks, the LMPE thus enabled not only offers general advantages of printed electronics but also displays many unique characteristics such as easy operation, no need for post-processing and diverse pervasive adaptability (Wang and Liu, 2016). Looking back to its research history, the first ever concept of LMPE can be traced back to the early days of this century in the present lab when developing liquid metal electronics cooling (Liu and Zhou, 2002; Ma and Liu, 2007b). At that time, when manipulating liquid metal, we often inadvertently splash liquid metal droplets to the nearby computer screen. The more we tried to scrub them off, the worse these droplets just stay there and even cause mush worse smearing. That was definitely not a pleasant experience at the initial. But with the time going on, it occurs to the senior author of this article that the scratched liquid metal residue might be adopted to make a circuit. This led to the conceptual incubation of LMPE. However, the subsequent years' trials unfortunately lasted a pretty long time. The reason lies in that liquid metal could not be easily written on a substrate without additional modifications. In another word, pure liquid metal or allied alloy is not a printable ink in strict sense due to its easy fluidity, high surface tension and poor compatibility with many materials. However, such fact was surprisingly not understood well at those old days. In fact, there exists a strong lack of liquid metal science and technology then. Fortunately, it was gradually realized that controllable adjustment of adhesion between modified liquid metal and substrates is the core issue for successful LMPE. And technological breakthrough was kept made around 2011 on the printing way (Liu and Li, 2011) and liquid metal adhesion on diverse substrates (Gao et al., 2012; Gao and Liu, 2012; Li et al., 2012). Such success suggests that the new generation dream ink for electronics printing is almost to come. Here, “dream ink” is in fact abbreviated from an academic term “DREAM Ink: Direct wRiting/pRinting of Electronics based on Alloy and Metal Ink” which late also leads to the setup of an LMPE company with the same name. Such a term implies that liquid metal does can serve as perfect dreamful electronic inks including not only electrical but also semiconductor or more functional inks (Liu 2012a, 2012b) which stimulated tremendous following studies. Such circuit can even be made as colorful (Liang et al., 2018) which would display pleasant esthetic feature or printed as transparent electronics (Mei et al., 2013; Zhang et al., 2014). So far, a series of intense investigations have been succeeded. With the time going on, more and more international labs are joining with many outstanding works reported in the area (Boley et al., 2014; Cao et al., 2020; Khondoker and Sameoto, 2016; Parekh et al., 2016; Ma et al., 2019; Markvicka et al., 2018; Silva et al., 2020; Teng et al., 2019; Yalcintas et al., 2019) which further significantly strengthened the research endeavor.

**Figure 2:**
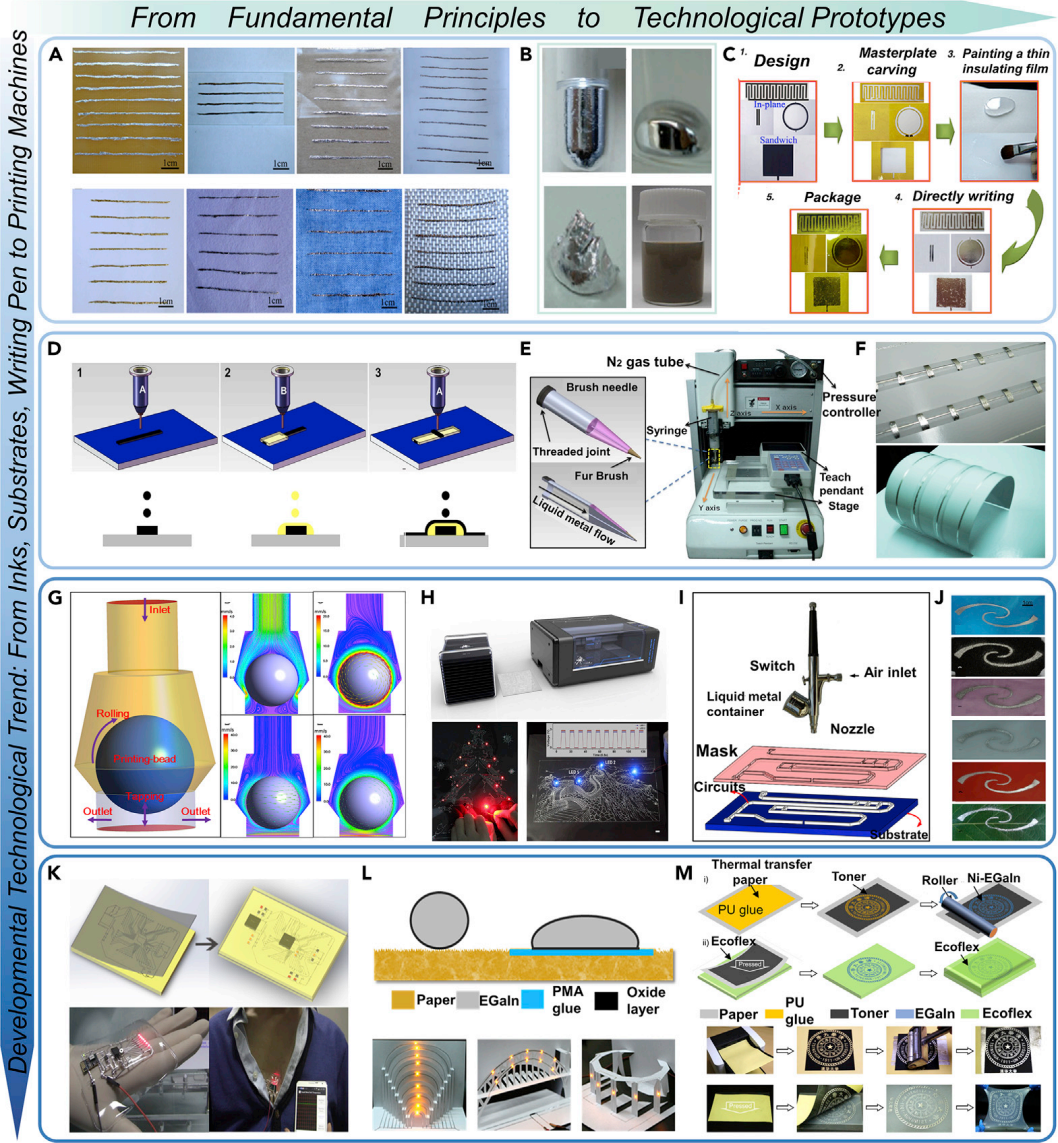
Basic principles and key technologies. (A) Adhesion-enabled liquid metal printed electronics. Demonstrated wettability of liquid metal ink written on different substrate materials. Images adapted with permission from Gao et al. (2012). (B) Pure liquid metal (top part) and liquid metal ink (bottom part). Images adapted with permission from Chechetka et al. (2017); Images adapted with permission from Gao and Liu (2012). (C) The direct writing process of patterned electrical components by using liquid metal ink. Images adapted with permission from Gao et al. (2013). (D) Schematic for hybrid 3D printing of liquid metal inks and packaging materials. Images adapted with permission from (Zheng et al., 2013a). (E) Lab prototype for desktop liquid metal 3D printer. Images adapted with permission from (Zheng et al., 2013a). (F) Fabricated electronic components with packaging layer via 3D hybrid liquid metal printing. Images adapted with permission from (Zheng et al., 2013a). (G) The composite fluid mechanics to deliver, transfer, and adhere liquid metal ink to the target substrate. Images adapted with permission from Zheng et al. (2014). (H) The commercial liquid metal printer and its quickly printed out lighting drawings. Images adapted with permission from Zheng et al. (2014). (I) Diagram for the rapid prototyping circuits based on atomized spraying printing of liquid metal droplets. Images adapted with permission from Zhang et al. (2014). (J) Liquid metal was demonstrated to be printable on any desired substrate surfaces via the atomized spraying printing. Images adapted with permission from Zhang et al. (2014). (K) Fabrication of flexible electronic device through dual-trans method to print first functional liquid metal circuit layout on PVC film and then transfer it into PDMS substrate via a freeze phase transition mechanism. Images adapted with permission from Wang et al. (2015). (L) One-step liquid metal transfer printing to fabricate flexible electronics on wide range of substrates. Images adapted with permission from Guo et al. (2018a). (M) Principle and practical working of SMART printing (Semi-liquid Metal and Adhesion-selection enabled Rolling and Transfer Printing). Images adapted with permission from Guo et al. (2019).

Due to the high surface tension issue (Daeneke et al., 2018), it is often challenging for liquid metal to wet many surfaces under normal conditions, thus making it difficult to achieve tight bonding between liquid metal and the target surface. Previously, the wettability of liquid metal-based electronic ink with other materials has not been improved fundamentally, which seriously restricts the practical value of such electronic ink. Based on the findings on the micro-oxidation strategy to enhance adhesion of liquid metal inks (Gao et al., 2012), it became a reality for the first time to lay electronics on various target substrates via only an ordinary pen or brush (Figures 2A and 2B). This therefore allows direct writing of electronic components on various target substrates (Figure 2C). It was disclosed that the addition of oxides would significantly increase the surface energy of the liquid metal ink, reduce the contact angle between the ink and the substrate and thereby improve the tendency for adhesion to occur as well as adhesion fastness (Gao et al., 2012; Gao and Liu, 2012). Further study offers more specific information on surface topography of the substrate to affect the wettability between liquid metal and substrate (Kadlaskar et al., 2017; Kramer et al., 2014). Considering that the conductivity of general liquid metal is still somewhat limited, the concept of nano liquid metal was proposed from which the nanoparticles mediated liquid metal materials' properties can be significantly enhanced (Ma and Liu, 2007a; Liu 2016). Along this direction, except for the oxidation modification, researchers also explored doping particles to modify liquid metal inks with expected adhesion capability. Such method effectively promotes the oxidation of liquid metal, as well as changing its fluidity. As designed, the liquid metal-particle composites exhibit desirable wettability to the substrate. Currently, successfully doped metal particles have included Cu, Ag (Tang et al., 2017), Fe (Cao et al., 2020; Carle et al., 2017; Hu et al., 2019), Ni (Chang et al., 2018; Guo et al., 2018c), Mg (Wang et al., 2018c), W (Kong et al., 2019), etc. Further, based on more generalized liquid metal composite strategy (Chen et al., 2020), the liquid metal dispersed into micro-nano droplets can form liquid metal-polymer composites with the polymer (Chechetka et al., 2017; Fassler and Majidi, 2015; Krisnadi et al., 2020; Li et al., 2018, 2020; Peng et al., 2019; Tang et al., 2018; Wang et al., 2018b), and the resulting ink can be flexibly applied to different substrates (Figure 2B). But because of the dispersed liquid metal droplets, special treatments to make them conductive are usually necessary, such as laser (Deng and Cheng, 2019; Liu et al., 2018a), low temperature (Chen et al., 2019; Wang et al., 2019), mechanical pressure (Boley et al., 2015; Zhang et al., 2019a), evaporation (Li et al., 2019b), *in situ* reduction of silver shell (Zheng et al., 2020), and stretch (Thrasher et al., 2019; Xin et al., 2019). Along with more inks and printing, liquid metal can be printed into 2D semiconductor (Lin et al., 2019) or applied to directly compose transistor and functional device (Li et al., 2019a).

From practical purpose, realizing large-scale automated manufacturing of LMPE is essential, so it is of great significance to invent equipment that can efficiently print liquid metal out. Through years' continuous endeavors, the present lab has made tremendous efforts in developing various potentially feasible methods to print liquid metal and managed to invent a group of very practical equipment. A noteworthy effort is that in the year of 2013, a big step toward truly direct LMPE was disclosed (Zheng et al., 2013a) which leads to the concept of personal electronics fabrications. Through making adhesive enough liquid metal inks, developing porous printing pinhead and innovating a prototyping machine among many different printing mechanisms (Figure 2D), as well as identifying matching paper among various substrate candidates, we demonstrated the first ever room temperature direct desktop LMPE machine (Figure 2E). In addition, through incorporation of the vulcanized silicone rubber as isolating inks, direct printing of a three-dimensional (3-D) multistory hybrid electro-mechanical device with encapsulated structure was also demonstrated (Figure 2F), while most of the former 3-D printings are only capable of making mechanical objects without electronics features inside. As a result, various typical circuits and functional components have been successfully printed out, including conductive wires, 3D conductor coil, and flexible antenna etc. And this basic electronic manufacture approach can be extended to more complicated three-dimensional situation. It could be noticed that, nearly all the above printing modalities including applications were repeated by a much late study (Park et al., 2019). Further, through introducing a tapping-mode composite fluid delivery mechanism (Figure 2G), a highly cost-effective and entirely automatic printing machine toward personal electronics fabrication was invented (Zheng et al., 2014). Such strategy integrates the fluid transport including up and down knocking ink feed, transfer printing, etc., thereby solving the problem that the liquid metal ink with high surface tension is difficult to drive smoothly through conventional ways. Via such an approach, the reliable printing, transfer and adhesion of the liquid metal inks on the substrate were achieved. This liquid metal printer is already close for an industrial use, and a series of electronic patterns spanning from IC, printed circuit board (PCB), electronic paintings, or do-it-yourself devices were demonstrated to be printed out with high resolution in a moment (Figure 2H). Also, the total engineering machine cost is highly friendly which paves the way for large scale personal electronics manufacture (Yang et al., 2015). In fact, a group of commercially available liquid metal circuit printers were thus produced which have now witnessed increasingly spreading use throughout the whole China.

With the above success in developing feasible electronics printing, a basic perhaps also ultimate question would naturally be raised that can we print liquid metal electronics on any desired surface? The answer is yes! As disclosed in a later study, Zhang et al. proposed and experimentally demonstrated a ubiquitous way of printing liquid metal on any target substrate surfaces via the atomized spraying principle (Figure 2I) (Zhang et al., 2014). This method has generalized purpose and is highly flexible and capable of fabricating electronic components on various objects, with either flat or rough surfaces, or made of different materials, or varied orientations from 1-D to 3-D geometrical configurations (Figure 2J). With a pre-designed mask, the liquid metal ink can be directly deposited on the substrate to form specifically designed patterns which lead to the rapid prototyping of electronic devices. Unlike the former direct writing technology, where large surface tension and poor adhesion between the liquid metal and the substrate often impede the flexible printing process, the liquid metal here no longer needs to be pre-oxidized to guarantee its applicability on target substrates. One critical mechanism was found as that the atomized liquid metal microdroplets can be quickly oxidized in the air due to its large specific surface area, thus resulting in a significant increase of the adhesive capacity and firm deposition of the ink to the substrate. This finding paved a generalized way for pervasively and directly printing electronics on various substrates which are expected to be significant in a wide spectrum of electrical engineering areas. An extended effort on the above method leads to the screen printing where liquid metal can be deposited via high pressure to realize high resolution complex electronic patterns (Wang and Liu, 2015b). Traditionally, the substrate materials suitable for LMPE mainly include a few materials such as polyvinyl chloride (PVC), PC, PI, while such newly emerging technology could adapt to many different substrates like glass, cloth, paper, skin, leaves and other arbitrary materials. Liquid metal inks can even be printed into three-dimensional self-healing hydrogels to compose soft electronics (Yu et al., 2017).

Owing to a group of efforts to tackle the fundamental issues and to fulfill various specific practical needs, researchers explored a variety of methods for preparing further powerful LMPE. Among them, a group of transfer printing methods with high manufacturing efficiency were especially fully explored and presented (Jiang et al., 2017, 2020; Ma et al., 2018; Wang et al., 2015; Wu et al., 2018b; Zhang et al., 2019b). The earliest trial along this direction is the proposition of the liquid metal dual-trans printing (Wang et al., 2015) which is based on the freeze phase transition mechanism of liquid metal as well as its transfer delivery (Figure 2K). Owing to the significant adhesion variation caused by the introduced freezing processing on the alloy, the liquid metal pre-printed on the PVC film can be transferred to the covering polydimethylsiloxane (PDMS), which overcomes the obstacles encountered before and works much simpler and faster to fabricate flexible and functional circuits encased in the elastic substrates. Further, Guo et al. (Guo et al., 2018a) proposed a one-step liquid metal transfer printing method with ubiquitous substrate adaptability (Figure 2L). The whole system comprised of polymer-based adhesive glue, its printing machine, the LMs ink, and the soft substrate, respectively. It was demonstrated that even on those substrates with weak wettability to LMs, the liquid metal transfer printing still works well to create complex conductive geometries, multilayer circuits and large-area conductive patterns with excellent transfer efficiency, facile fabrication process and remarkable electrical stability. This suggests a universal way for wide spread practices of liquid metal enabled soft and flexible electronics. In fact, the selective printing method has plenty of space to explore. Although combining the rolling and transfer printing together, Guo et al. (Guo et al., 2019) presented the so called SMART Printing which is abbreviated from the term “Semi-liquid Metal and Adhesion-selection enabled Rolling and Transfer Printing” (Figure 2M). Based on the semi-liquid metal and its adhesion-difference on specifically designed target materials, it was shown that the SMART printing could serve to rapidly manufacture a wide variety of complicated electronic patterns with high resolution and large size. The process is much faster than most of the currently existing electronic fabrication strategies, say just a few seconds. The above method has generalized purpose. Many different adhesion materials such as superhydrophobic coating or the like can be introduced to realize selective transfer LMPE (Yao et al., 2019). And multiple times' transfer was also proposed and demonstrated to fabricate 3D soft electronics via a rather quick, easy going and low cost way (Zhao et al., 2020).

It should be pointed out that, up to now, most of the above key technologies ever developed have been or are being translated into practicing which favorably promoted the progress of LMPE industry. Clearly, in the coming time, new technologies enabled from LMPE are still emerging. All these efforts built a bridge from laboratory to industry, which significantly speed up the rapid industrialization of LMPE. Clearly, the principle breakthrough and technological accumulation in the early stage are prerequisites to practical application of corresponding instruments, industrialization of room temperature liquid metal printing. To a large extent, such endeavors have broken the technical bottlenecks and barriers of personal electronic circuit manufacturing, making it a reality to fabricate electronic circuits quickly at will, especially for flexible electronics. Until now, with the tremendous trials and solid foundation thus laid, the industrialization of LMPE was significantly stimulated which led to the setup of several commercial companies around the year of 2013 and a group of commercial products have been available in market indeed.

## Commercial product development and industrialization road map

**Figure 3:**
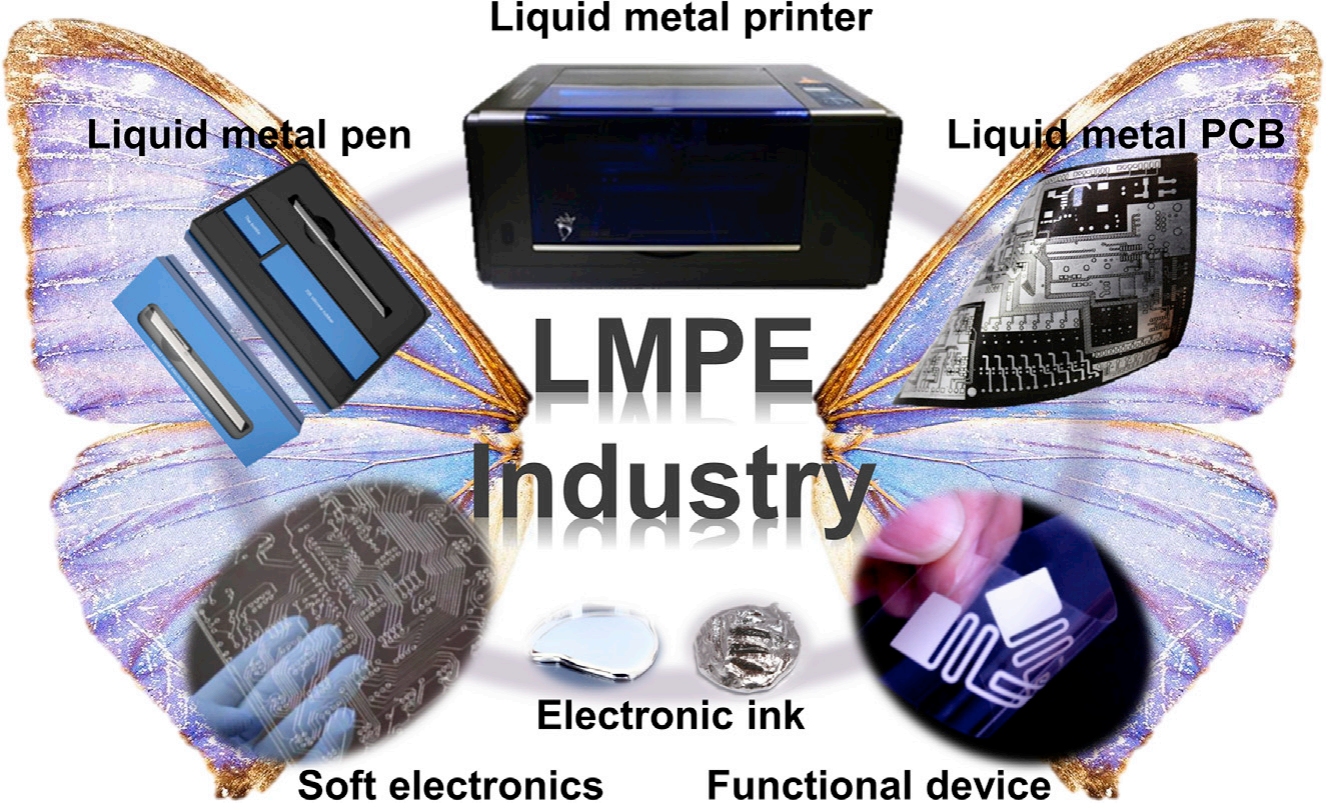
Typical products based on liquid metal printed electronics give wings to its industrialization (LMPE Industry)

Industrialization plays diverse vital roles including strengthening fundamental research, especially applied science. It is in fact an ultimate goal of scientific research. To achieve industrialization, one needs first to clarify the fields and directions where LMPE can be best applied, and corresponding products that well meet market should be invented. Ideally, any practical situations that can replace traditional electronics manufacturing methods with printing can be replaced by LMPE technology. This thereby indicates an enormous market prospect. The most direct application example is the replacement of printed circuit board (PCB) manufacturing process with LMPE technology. So far, the classical ways of making printed circuit boards generally need cumbersome steps while LMPE technology completely avoids such tedious procedures. Currently, LMPE technology can be used to realize real-time production of single-layer and double-layer circuit boards, providing convenient and efficient basic tools for complex circuit design and verification, and creating excellent conditions for improving product iteration cycles. Hence, relevant LMPE products will provide a boost to the development of industrialization. As illustrated in Figure 3, a group of core products and applications aimed for market have been continuously put forward, including but not limited to liquid metal electronic inks, printing substrates and packaging materials, liquid metal electronic writing pens, electronic circuit printers, functional devices, etc.

These products and applications exhibit evident advantages over existing ones. For example, the circuit pattern can be directly formed by portable electronic pen, which efficiently avoids the corrosion and shortens the production process, thereby guaranteeing environmental protection. Moreover, the emergence of highly automated liquid metal electronic circuit printer makes it possible to write out functional circuits quickly on different substrates without needing complicated post-processing. Such technology makes the direct preparation of electronic devices no longer an unattainable goal. Until now, researchers have already been able to directly produce themselves antennas (Lin et al., 2015), RFID tag (Gao et al., 2016), capacitors (Gao et al., 2013), and many more other devices (Kim et al., 2020). Therefore, it can be seen that LMPE is opening a huge application space, especially in personalized electronic circuit manufacturing. In the near future, LMPE can be extended to nearly all electrical engineering particularly in smart wearables, skin electronics, flexible sensing, biomedicine and other fields, while also providing unique and effective tools for the education and artistic creations. It is expected to see more excellent products or applications based on LMPE technology, which will provide wings for the take-off of the LMPE industry, just as reflected by the image of Figure 3. Besides, although the liquid metal-based series of products were put into production, the promotion, and application of the products have been evidently hindered due to lack of relevant national standards for liquid metal, which has also become an obstacle to the rapid development of liquid metal industry. In order to solve such problem, tremendous efforts from both academia and industry were ever made toward drafting a series of industrial standards for liquid metal since 2017. Among them, the national standard of “Gallium-based Liquid Metal” has finally got approved after nearly two years of continuous work and will be officially released in the early 2021 (http://www.cnsmq.com/index.php?m=content&c=index&a=show&catid=26&id=3020). This is expected to be conducive to the canonical development of the liquid metal industry and will significantly promote the expansion of related application fields.

**Figure 4:**
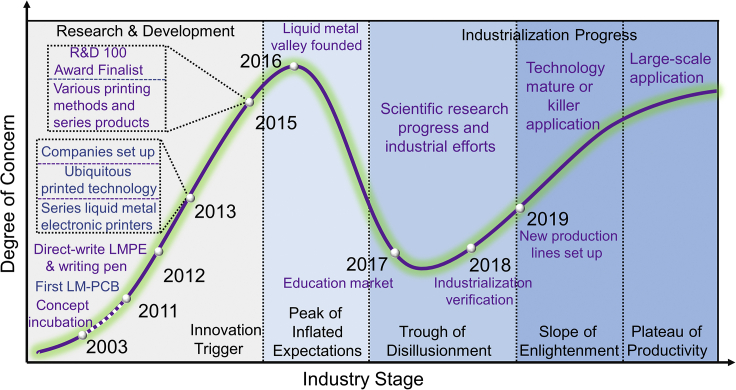
Industrialization stage of liquid metal printed electronics

Because of the emergence of products that cater to market demand, the LMPE industry is developing steadily. Learning from the past, screening the entire advancing stage of LMPE from laboratory to industrialization is an insightful thing. For clarity, we draw a developmental diagram of the LMPE industry, which is not only to summarize the past but also to guide further road map. As illustrated in Figure 4, the incubation of LMPE can be traced back to 2003 (Liu and Zhou 2002) and official prototype machine appeared in 2011 (Liu and Li, 2011). Since then, a series of breakthroughs have been continuously achieved. Among them, typical technologies include direct writing printed electronics device realized in 2012 (Gao et al., 2012; Li et al., 2012). Further, multiple liquid metal electronic printers to fulfill various printing tasks were also successfully achieved, and related companies were established in the year of 2013 (Zheng et al., 2013a, Zheng et al., 2013b). Two years later, more printing methods and products appeared. Meanwhile, LMPE printer won the 2015 R&D 100 Award Finalist, indicating that it has received extensive attention over the world industry. The above can be regarded as the innovation trigger over the industrialization of LMPE. In this stage, a serial of technological breakthroughs attracted extensive attention, and their industrialization had ever been placed with high expectations. After that, the highest level of attention to LMPE industry appeared in 2016, when the long-expected liquid metal valley was established while its earliest proposition was in fact put forward at around 2008.

However, just as frequently happened in history, industrialization is always a systematic and challenging thing, which means that unexpected obstacles will inevitably be encountered in its developmental process. As indicated in Figure 4, after the stage of innovation trigger, the stage of “the peak of inflated expectations” appears, in which extraordinary expectations for the prospect of industrialization reached its peak along with the release of a huge number of scientific news reports on LMPE throughout the world (https://www.technologyreview.com/2013/11/19/112693/). Then, owing to the unanticipated barriers and unsmooth industrialization process, the public's attention may drop down, which leads industrialization to enter the stage of “trough of disillusionment”. This stage is an important test of the industrialization prospects of new technologies, and many start-up companies unfortunately fail at this stage. Inspiringly, liquid metal has gained attention and recognition in the education market because of its unique advantage of WYSIWYG (What You See Is What You Get), which provides strong support for the steady growth of its subsequent industrialization. Later, thanks to continuous scientific research and successful industrialization verification, slope of enlightenment of LMPE industry was successfully realized. Until this stage, LMPE-based products become more abundant and competitive. Besides, the related market is further expanded, and new production lines to increasingly meet various market demands have been established. With the application of more mature technologies and the further development of killer application, the large-scale practice of LMPE will soon be expected. Finally, with its successful industrialization, LMPE will also re-assume its high attention again from the public. Such a development trend and deemed roadmap will help inspire the society to overcome the obstacles encountered in each stage in order to achieve the coming large-scale industrialization. To do this, giving full play to the important role of all factors is rather essential.

**Figure 5:**
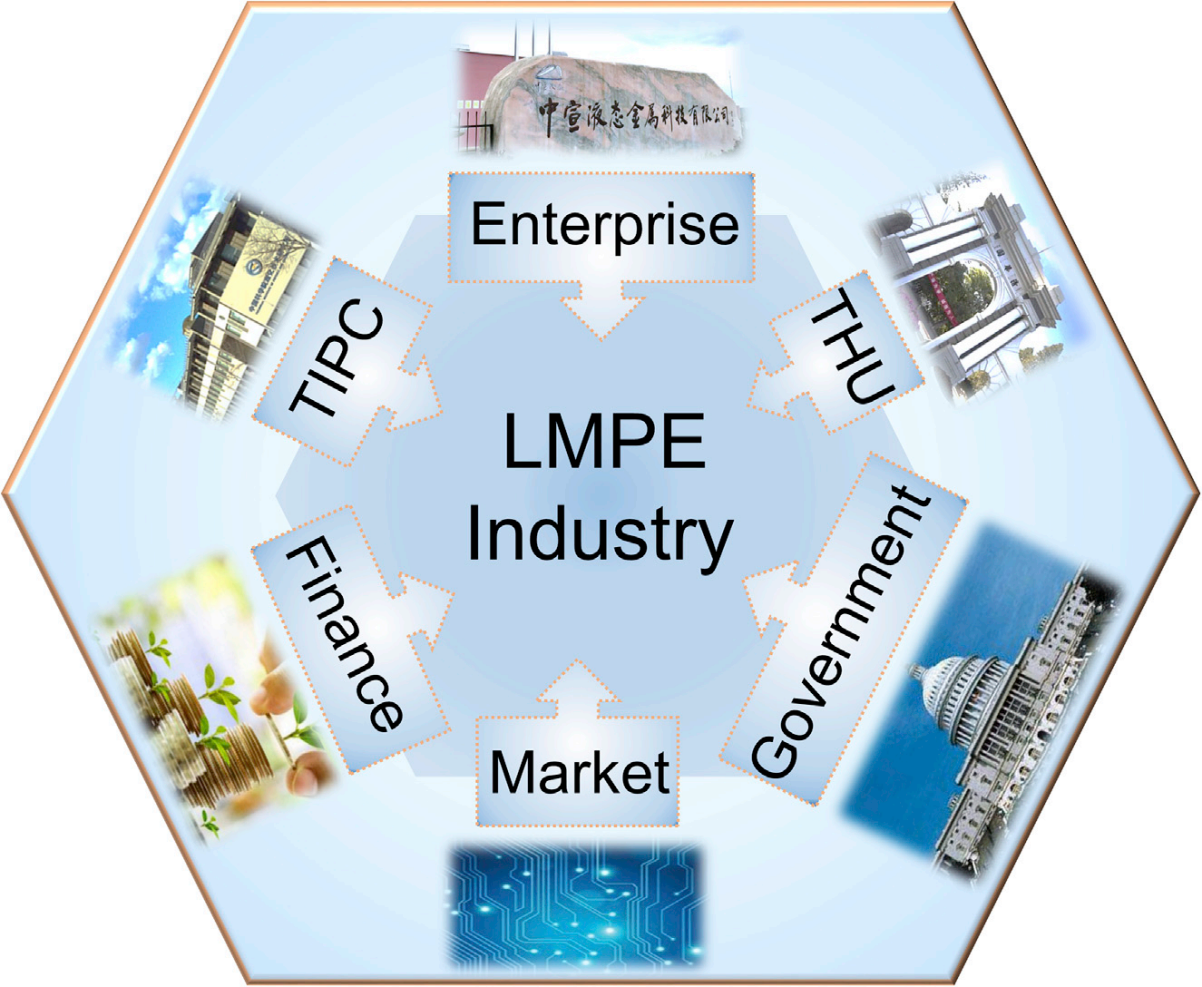
Important factors affecting the development of the LMPE industry Here, THU indicates the Tsinghua University, and TIPC stands for Technical Institute of Physics and Chemistry of the Chinese Academy of Sciences.

Further, we summarize important factors affecting the development of industrialization. First, the cost of the product is not negligible for the development of any industry. Currently, the price of Gallium-based liquid metal is about a quarter of the price of silver, which seems to be quite high. However, the price of the final product is affected by many factors, including not only the price of raw materials, but also the product performance, competitiveness, market demand, etc. Therefore, maintaining a reasonable product price requires comprehensive consideration of many factors. In addition to cost, more factors are affecting the development of LMPE industry (Figure 5). For LMPE industry, universities (THU) and academic institutions (TIPC) provided the initial technology for industrialization, which can be regarded as the foundation of industrialization. Profit-making enterprises serve as the structural main body of industrialization, whose purpose is to continuously improve efficiency and reduce costs driven by profit mechanisms. Therefore, we must establish and perfect the current corporate system, form a technological innovation mechanism, and improve technological innovation capabilities, so as to give full play to the pioneering role of enterprises in industrialization. The role of the government in the process of industrialization is mainly reflected in the policy-oriented role, organizational coordination and service role. Although this role of the government is not decisive, it is still critically important, especially for this kind of start-up brand new industry. In addition, financial support is also quite necessary, which is not only related to the smooth establishment of new industry, but also conducive to its sustained and rapid development in the future. The market is the starting point and ending of transforming scientific and technological achievements into commodities, which determines the success of industrialization. Market demand and competition promote the continuous development of products. Only advanced products that meet the market can achieve industrialization success. For a new industry, it is extremely important to continuously tap the market demand and open up new markets.

These factors run together to form a joint force, which will help promote the rapid development of LMPE industrialization. In addition, there is also an interest relationship between them. To take advantage of such benefits, it is vital to deal with the relationship between various interests, personal gaming and collective outputs, as well as the relationship between short-term interests and long-term profits in the process of industrialization. To a large extent, handling the interests of all parties involved in industrialization is a prerequisite for the sustained development of industrialization.

## Discussion and prospects

Overall, the major features and advantages of LMPE are targeted for pervasively personal use, large-area printing, flexibility, low cost, environmentally friendly and high performance, which is ultimate goal of human being however in sharp contrast with silicon-based microelectronics. The specialty of LMPE technology lies in a brand new set of electronic preparations via low-temperature additive manufacturing. Such strategy revolutionizes the traditional manufacturing concept of electronic engineering. Its WYSIWYG (What You See Is What You Get) modality in electronic printing opens a realistic way to develop exclusive electronic fabrication technology and reshapes conventional electronics and IC manufacturing rules. Besides, owing to the unique merits of LMPE technology, it is especially applicable to an inexperienced user. That means, anyone can freely adopt LMPE machine to make their own circuit. This thereby would significantly accelerate the arrival of the personalized electronics manufacturing era. Clearly, such impact will not only happen in the industry but will also be widely reflected in the fields of education, design, cultural creativity, etc.

**Figure 6:**
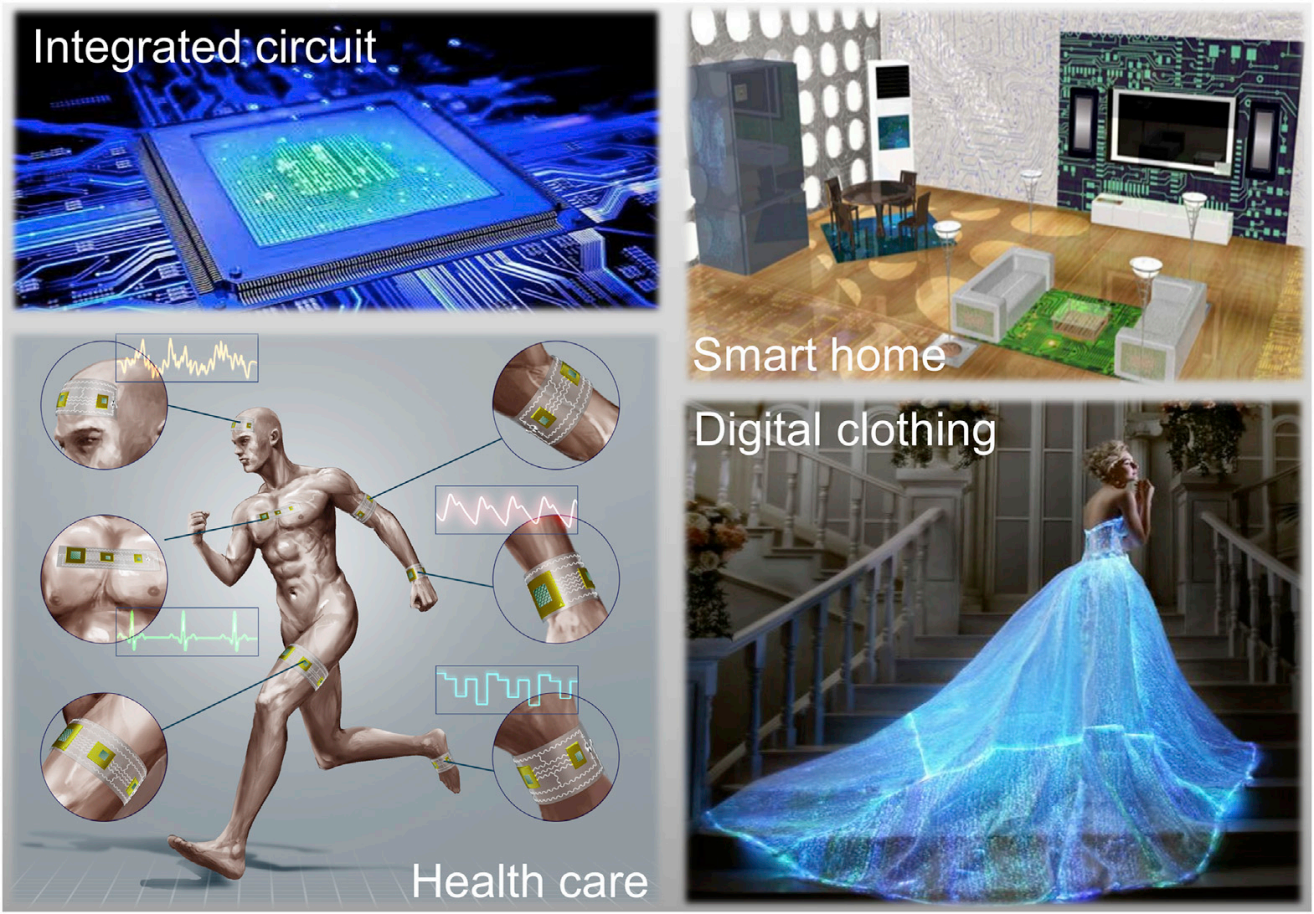
Several typical application scenarios of LMPE Examples are LMPE enabled integrated circuit, smart home equipped with various liquid metal printed electronics here and there, human health care and smart clothing made of liquid metal printed electronics. Images adapted with permission from Guo et al. (2018b).

Clearly, LMPE can be adapted to various substrates and compatible with a variety of materials, thus forming a large number of application areas, such as organic electronics, plastic electronics, flexible electronics, transparent electronics, paper electronics, skin electronics, wearable electronics, etc. In the near future, it can be expected that LMPE would display ever broad application prospects. To name just a few of them, Figure 6 illustrates several practical categories for the administration of LMPE such as smart home (Wang and Liu, 2015a), skin electronics for human health care, digital clothing, and more.

At present, more and more laboratories in academia and industrial companies around the world are joining the corresponding R&D and industrial promotion, and a new liquid metal electronics industry is quietly taking shape. Fortunately, the current market for flexible electronics is huge. According to reliable statistics and forecasts, the market for flexible electronics will reach US$300 billion in a few years (https://www.idtechex.com/). Consequently, it is believed that the LMPE industry will develop rapidly in the future. Surely, knowledge and technological innovation mainly come from scientific research. According to the industrial developmental roadmap, further breakthroughs in scientific research will help the industry evolve to a new stage. In this sense, some unsolved scientific issues or challenges related to LMPE technology urgently need to be tackled for achieving the expected rapid development of the LMPE industry.

The first part is improving the resolution and quality of the manufactured electronic circuits. So far, the obtained liquid metal electronic circuit printer has reached the level of writing wires in tens of microns width. Further exploration along this road is vital for the practice of LMPE in the field of microelectronics. As discussed before, the micro-oxidation strategy can effectively improve the wettability between the liquid metal and the substrate, but oxidation also damages its own high conductivity. Therefore, finding new strategies for direct printing without losing inks' performances is highly meaningful. Besides, proper packaging is important for the practice of electronic circuits. Tremendous reliable tests and quick ways for high quality fabrication are yet to complete. Also, the long-term stability and durability of products is crucial, and there is still a lot of work to be done about the toxicity of liquid metal under ultra-long-term use and the aging test under various application conditions.

Regarding the electronic inks, the liquid metal materials genome project as raised in (Wang and Liu, 2013) would help find more liquid metal candidates with better properties in the future. Meanwhile, it is necessary to explore recyclable strategies in many aspects including reducing costs. Also, LMPE is directly connected with flexible electronics and the liquid metal with excellent fluidity allows the electronic circuit thus made maintain outstanding conduction during various deformation processes such as bending and stretching. This brings enormous opportunities to the field of flexible electronics. Moreover, liquid metal ink can be combined with different semiconductors to directly write out ending use device (Li et al., 2019a). This means, pervasively available ICs and functional products can finally be directly printed out in the coming time.

### Conclusion

Given the electronics industry is one of the largest over the world, LMPE clearly exhibits an extraordinary big market prospect. Tremendous opportunities are in incubation. The key toward industrialization is to find a suitable entry point (e.g. flexible PCB), which can not only reflect the superiority of the direct printing but also encourage profound market needs. In the long run, LMPE can also inspire and create new markets. This is because such new generation technology’s unique merits in large-area printing, flexibility, and environmental friendliness of the printed electronic products would foster new consumer demand. It is expected that the LMPE industry will eventually witness an ever exploded prosperity with the putting forward of the scientific and technological achievements into thousands and millions of households until benefiting all walks of life.

## Limitations of the study

The description of the factors affecting the industrialization of LMPE in Figure 5 is general, and more quantitative effects are difficult to obtain.

## Resource availability

### Lead contact

Further information and requests should be directed to and will be fulfilled by the lead contact, Jing Liu (jliu@mail.ipc.ac.cn)

### Materials availability

This study did not generate any new materials.

### Data and code availability

Any data utilized in this study can be found in the main manuscript.
